# Optical imaging featuring both long working distance and high spatial resolution by correcting the aberration of a large aperture lens

**DOI:** 10.1038/s41598-018-27289-1

**Published:** 2018-06-15

**Authors:** Changsoon Choi, Kyung-Deok Song, Sungsam Kang, Jin-Sung Park, Wonshik Choi

**Affiliations:** 1 Center for Molecular Spectroscopy and Dynamics, Institute for Basic Science, Seoul, 02841 Korea; 2 Department of Physics, Korea University, Seoul, 02841 Korea

## Abstract

High-resolution optical imaging within thick objects has been a challenging task due to the short working distance of conventional high numerical aperture (NA) objective lenses. Lenses with a large physical diameter and thus a large aperture, such as microscope condenser lenses, can feature both a large NA and a long working distance. However, such lenses suffer from strong aberrations. To overcome this problem, we present a method to correct the aberrations of a transmission-mode imaging system that is composed of two condensers. The proposed method separately identifies and corrects aberrations of illumination and collection lenses of up to 1.2 NA by iteratively optimizing the total intensity of the synthetic aperture images in the forward and phase-conjugation processes. At a source wavelength of 785 nm, we demonstrated a spatial resolution of 372 nm at extremely long working distances of up to 1.6 mm, an order of magnitude improvement in comparison to conventional objective lenses. Our method of converting microscope condensers to high-quality objectives may facilitate increases in the imaging depths of super-resolution and expansion microscopes.

## Introduction

The performance of an optical microscope is often characterized by its spatial resolving power. In practice, however, the working distance of objective lenses may also be an important factor when objects of interest are located well below a surface. In general, the working distance tends to be reduced as the numerical aperture (NA) of the objectives determining the spatial resolving power increases. For example, the working distance of 0.4 NA dry type objectives can be as long as 3.9 mm while that of 1.4 NA oil-immersion type objectives is only around 200 μm. Manufacturing an objective that has both long working distance and high NA requires a physically large diameter lens to secure long working distance and yet its focal length should be short for high NA. However, designing and fabricating such a large diameter lens with an ideal point-spread-function (PSF) is difficult since such a bulky lens is likely to introduce severe aberration. This problem has fundamentally limited the imaging depth of high-resolution optical microscopes. For example, super-resolution microscopes such as STED, PALM, and STORM microscopes require aberration-free high NA objectives, but oil-immersion objectives can only meet this with short working distance^1–5^. Another interesting example is expansion microscopy that makes use of the additional physical magnification by expanding the volume of the samples by chemical processes^6–9^. While this approach can gain spatial resolution by the sample expansion, it simultaneously reduces effective working depth by the same factor of the resolution enhancement.

One of the most effective approaches to simultaneously achieving both high spatial resolution and long working distance is using aperture synthesis. In this approach, images taken by low NA objectives, through either coherent^10–16^ or incoherent^17–24^ measurements, are merged to form images equivalent to those taken by high NA objectives. However, it cannot lead to the physical form of a sharp focus at the sample plane because it applies post-processing to increase image resolution. Therefore, this method is hardly usable with the fluorescence-based scanning microscopes widely used in life science. Another potentially effective approach, one considered in the present study, is to use lenses with a physically large diameter and a short focal length. For this approach, commercial microscope condensers are good candidates. For example, the working distance of oil-immersion type condensers is an order of magnitude longer than that of the typical objective lenses with the same NA. However, as mentioned above, the critical drawback of using lenses such as condensers for imaging is their strong aberration. Aberrations tend to vary steeply with respect to the spatial frequency, particularly at high frequencies, which significantly degrades spatial resolution. Thus, correcting the aberration of large aperture lenses is essential for the development of microscopes featuring both long working distance and high spatial resolution.

Various techniques have been proposed in the field of adaptive optical microscopy for the correction of both system and sample-induced aberrations. Adaptive optical methods make use of spatial light modulators or deformable mirrors to control the wavefront of illumination and/or detection beams. Depending on the type of aberration measurement, these methods can be classified into direct^25–28^ and indirect^29–33^ wavefront sensing schemes. These methods have proven to be effective in various applications. For example, the adaptive optics was applied to the super-resolution techniques such as STED and STORM to acquire extremely high-resolution images from samples inducing aberrations^34–37^. Most such schemes are effective at identifying sample-induced aberrations and designed to work at high speed using a relatively small number of aberration correction elements in wavefront shaping devices. Therefore, they are poorly suited to coping with steep variations in aberrations spanning the entire pupil because this will require a large number of aberration correction elements. We recently developed an aberration correction approach employing indirect wavefront sensing methods called closed-loop accumulation of single scattering (CLASS) microscopy working in the reflection geometry^38^. This method measures and corrects sample-induced aberration by maximizing the total intensity of synthetic aperture images. One important feature of CLASS microscopy is that the angular resolution of the aberration measurements can be extremely high because the method relies on finely angularly scanned images rather than feedback control by the wavefront shaping devices for the correction of aberrations. Thus, it is well suited to dealing with the steep angular variations in aberration associated with the lenses with a large diameter.

In this study, we constructed a transmission microscope composed of two commercial microscope condensers and applied the CLASS algorithm to identify and numerically correct the aberrations of the individual condensers. The innate aberrations of these lenses cause waves containing high spatial frequency components of the object spectrum to be inaccurately accumulated. Consequently, resolving power is attenuated, and PSF is significantly broadened in comparison with ideal diffraction-limit PSF. The CLASS algorithm was applied to correct such strong aberrations by maximizing the total intensity of the synthetic aperture image. By performing the maximization operation on both forward and phase-conjugated waves, the aberration maps of the two condensers could be distinguished. At a source wavelength of 785 nm, we demonstrated wide-field transmission imaging with a spatial resolution of 372 nm to the extremely long working distance of 1.6 mm. The performance of the proposed method was evaluated when imaging both a resolution target and yeast cells. Our method will help to improve the working depth of super-resolution microscopes, which require high NA objectives for maximal performance.

## Experimental setup and data acquisition

**Figure 1:**
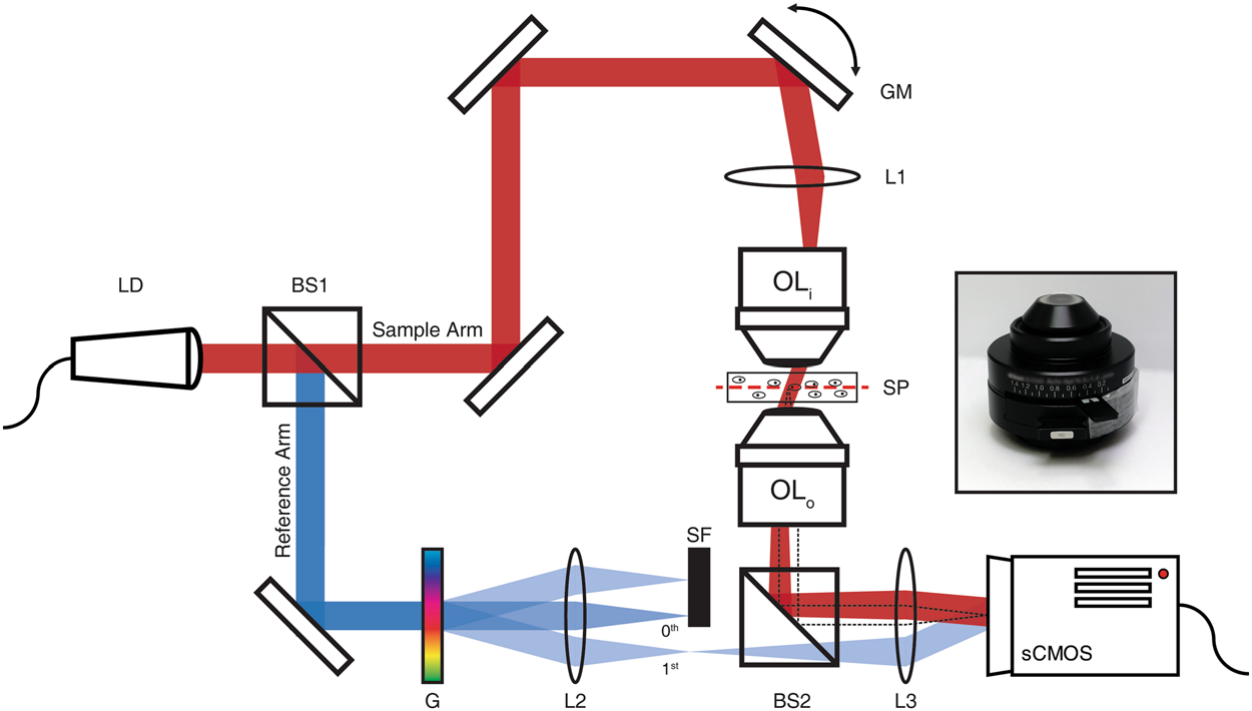
Schematic diagram of a transmission-mode off-axis interference microscope. A pair of oil-immersion type condenser lenses (OL_i_ and OL_o_) shown in the inset were used as objective lenses. Samples were placed between the two condensers. LD: laser diode, BS1-2: beam splitters, GM: 2-axis galvanometer mirror, L1-3: lenses, OL_i_: condenser for input (illumination), SP: sample plane, OL_o_: condenser for output (detection), G: grating, SF: spatial filter, sCMOS: camera.

We constructed a transmission-mode coherent imaging system based on a Mach-Zehnder interferometer (Fig. 1). To physically guarantee both long working distance and high spatial resolution, we employed two microscope condensers (MBL78700, Nikon; NA: 1.4, working distance: 1.6 mm) as illumination and imaging objective lenses. In this configuration, since the working distance of each condenser was 1.6 mm, the space between the two condensers where the sample could be inserted was 3.2 mm thick. A laser diode (LP785-SF100, Thorlabs; wavelength: 785 nm) was used as a light source. The output beam from the laser was split into the sample (red) and reference (blue) beams at the beam splitter (BS1), and the direction of illumination of the sample beam was controlled by a pair of galvanometer mirrors (GV011/M, Thorlabs). The sample beam was then introduced to the sample plane (SP) through an input condenser (OL_i_), and the wave transmitted through the sample was captured by the output condenser (OL_o_). The sample image was relayed to a scientific complementary metal oxide semiconductor (sCMOS) camera (pco.edge 4.2, PCO). The angle of the reference beam was tilted by selecting the first-order diffraction from a diffraction grating (G) with a spatial filter (SF), and then combined with the sample beam at the camera to acquire off-axis interference images at the camera. A 2D Hilbert transform was applied to the recorded interferograms to obtain the complex field maps, i.e., the phase and amplitude maps, of the transmitted waves^39,40^. The overall magnification from the sample plane to the camera was 80× and the view field was 50 × 50 µm^2^.

With a target in place, we took a set of complex field maps of transmitted waves, ${E}_{o}(x,y;{\overrightarrow{k}}^{i})$, by varying the illumination angle, or the transverse wavevector ${\overrightarrow{k}}^{i}$ of the incident wave. The ${\overrightarrow{k}}^{i}$ was chosen in such a way to cover all the orthogonal free modes in the given field of view and the numerical aperture. For the view field of 50 × 50 µm^2^ and the numerical aperture of 1.2, the required number of images amounted to19,861 images. Typical image acquisition rate was 20 fps in the present study such that the total data acquisition takes approximately 15 minutes. The image acquisition time can potentially be reduced by using a high-speed camera and by minimizing the number of images required for the aberration correction. We took Fourier transform of each recorded image with respect to the spatial coordinate (*x*, *y*) and obtained the spatial frequency spectrum ${ {\mathcal E} }_{o}({\overrightarrow{k}}^{o};{\overrightarrow{k}}^{i})$ of the transmitted wave. Here ${\overrightarrow{k}}^{o}$ is the transverse wavevector of the transmitted wave conjugate to (*x*, *y*). We used the measured ${ {\mathcal E} }_{o}({\overrightarrow{k}}^{o};{\overrightarrow{k}}^{i})$ to identify the system aberration and to reconstruct a target image (see details in the following sections).

## Effect of aberration on synthetic aperture imaging

**Figure 2:**
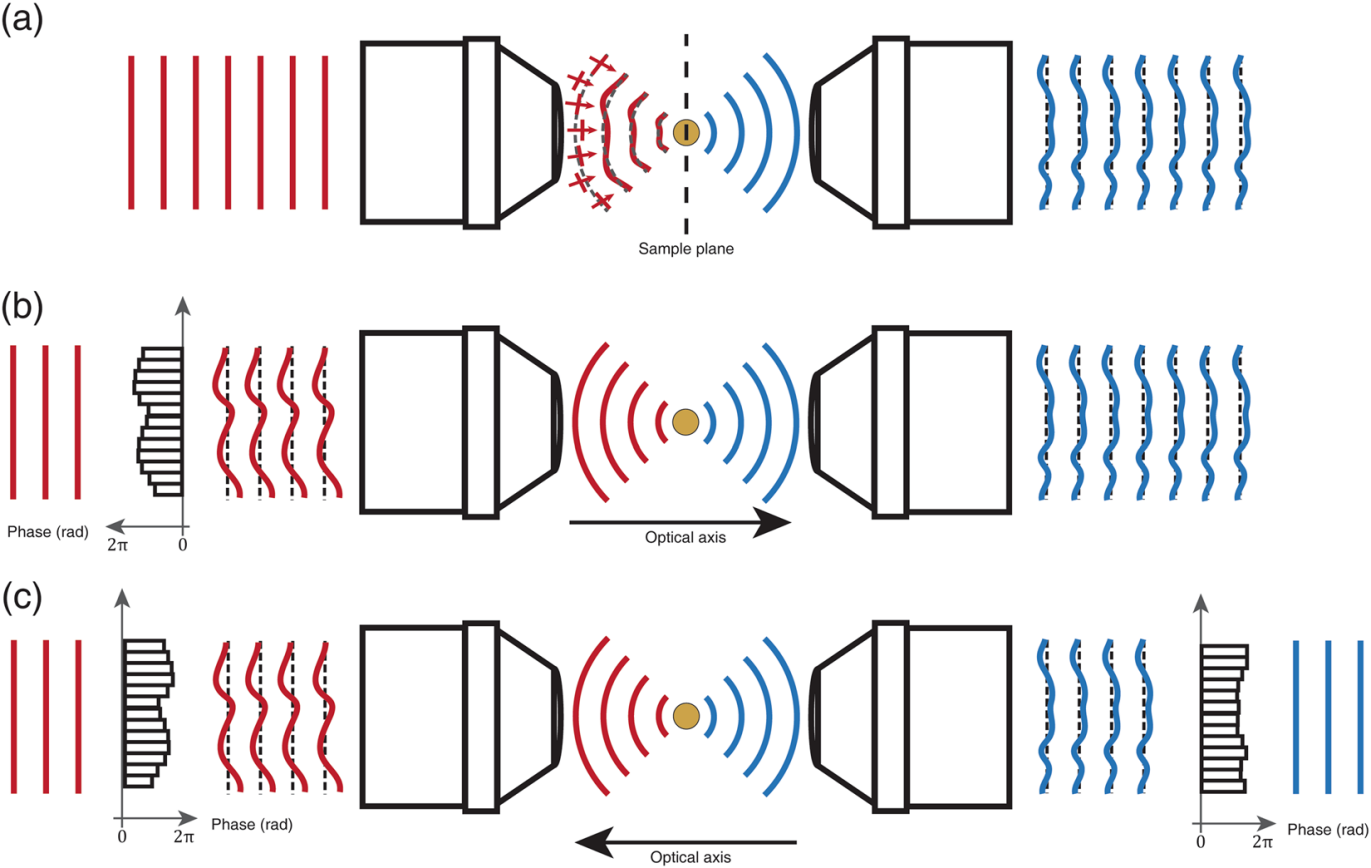
Principle of closed-loop accumulation of single scattering (CLASS) algorithm in the presence of system aberration. (**a**) A plane wave (red solid lines) is illuminated on the pupil of the input condenser lens. Due to condenser aberration, the plane wave at the sample plane (red curved lines) acquires a different phase shift. This yields a blurred and distorted PSF. In the transmission side (blue curves), the ideal point source appears to be a distorted plane wave after transmitting through the output condenser lens. (**b**) By placing a virtual SLM at the Fourier plane to compensate for the additional phase shift at different angles (graph with black bars), the back aperture of the input condenser is illuminated by a distorted plane wave. Due to this initial phase shift, the light focused at the sample plane forms a clean PSF. (**c**) Here, the direction of propagation (optical axis) is reversed numerically by phase-conjugation. In a reversal of the previous process, a virtual SLM (graph with black bars on the right) is placed at the output pupil to focus light at the sample plane to compensate for output aberration.

The condenser lens exhibits strong aberration, especially at large incidence angles. To describe the detrimental effects of this aberration on the formation of the image, consider a unit-amplitude plane wave $E(x,y,z=0;{\overrightarrow{k}}^{i})=\exp [i({k}_{x}^{i}x+{k}_{y}^{i}y)]$ at the sample plane, whose transverse wavevector is ${\overrightarrow{k}}^{i}\,=\,({k}_{x}^{i},{k}_{y}^{i})$. This plane wave propagates from the light source to the target object through the illumination condenser OL_i_. Aberration along this illumination pathway can be described by an input pupil function ${P}_{i}^{a}(\overrightarrow{k})=|{P}_{i}(\overrightarrow{k})|\exp [i{\varphi }_{i}(\overrightarrow{k})]$. Here, the function ${\varphi }_{i}(\overrightarrow{k})$ describes the phase retardation of the plane wave as a function of the transverse momentum $\overrightarrow{k}$. For an ideal lens, the pupil function ${P}_{i}$ will be unity for $|\overrightarrow{k}|\le {k}_{0}\alpha$, where ${k}_{0}$ is the magnitude of the wavevector in free space and $\alpha$ the NA of the lens, or zero otherwise^41^. In the presence of aberration, however, the plane wave may experience additional phase retardation depending on its propagation direction. Due to this aberration, the plane wave impinging on the pupil plane of OL_i_ cannot form a clean focus at the sample plane, as indicated by the red curves in Fig. 2(a). Similarly, the optical transfer function from the target object through OL_o_ to the detector can be described by an output pupil function ${P}_{o}^{a}(\overrightarrow{k})=|{P}_{o}(\overrightarrow{k})|\exp [i{\varphi }_{o}(\overrightarrow{k})]$. Here, the phase term ${\varphi }_{o}(\overrightarrow{k})$ can be regarded as the aberration induced by OL_o_. Due to this output aberration, the light scattered from a point target at the sample plane forms a distorted plane wave at the pupil plane of OL_o_ (blue curves in Fig. 2(a)). Our analysis assumes no loss at the pupil planes throughout, i.e. $|{P}_{i}|=|{P}_{o}|=1$ for $|\overrightarrow{k}|\le {k}_{0}\alpha$.

The effect of aberration from OL_i_ to SP is relatively straightforward as the incident wavevector ${\overrightarrow{k}}^{i}$ does not change direction. On the other hand, the effect of the output aberration is more complicated since the incoming wave to SP experiences alteration of the wavevector due to the target object. Let us consider the target object whose object spectrum is written as ${\mathscr{O}}(\Delta \overrightarrow{k})$. Due to the conservation of momentum, the transverse wavevector of the wave transmitted through the target object is modified to ${\overrightarrow{k}}^{o}={\overrightarrow{k}}^{i}+\Delta \overrightarrow{k}$ for each spatial frequency component $\Delta \overrightarrow{k}$ of the target object. For each plane wave illumination, which is a Dirac delta function in Fourier space, the angular spectrum of the scattered field will simply be the translation of the object spectrum by ${\overrightarrow{k}}^{i}$, which can be written as ${\mathscr{O}}({\overrightarrow{k}}^{o}={\overrightarrow{k}}^{i}+\Delta \overrightarrow{k})$. Therefore, the angular spectrum of the transmitted wave is written as

1 $${ {\mathcal E} }_{o}({\overrightarrow{k}}^{o}={\overrightarrow{k}}^{i}+\Delta \overrightarrow{k};\,{\overrightarrow{k}}^{i})={P}_{o}^{a}({\overrightarrow{k}}^{i}+\Delta \overrightarrow{k}){\mathscr{O}}({\overrightarrow{k}}^{i}+\Delta \overrightarrow{k}){P}_{i}^{a}({\overrightarrow{k}}^{i})\exp [ig({\overrightarrow{k}}^{i})].$$

Here $g({\overrightarrow{k}}^{i})$ is the uncontrolled phase drift occurring during image acquisition. The phase drift originates from fluctuations in the optical path length difference between the sample waves and reference waves over the multiple measurements taken when scanning ${\overrightarrow{k}}^{i}$.

The synthetic aperture image can be computed by demodulating the target spectra from the illumination wavevector. In other words, the angular spectrum of the synthetic aperture image can be obtained by adding the electric fields of the transmitted waves that have the same momentum difference ${\rm{\Delta }}\overrightarrow{k}$ acquired for various ${\overrightarrow{k}}^{i}$:

2 $${ {\mathcal E} }_{SA}({\rm{\Delta }}\overrightarrow{k})=\sum _{{\overrightarrow{k}}^{i}}{ {\mathcal E} }_{o}({\overrightarrow{k}}^{i}+{\rm{\Delta }}\overrightarrow{k};{\overrightarrow{k}}^{i})={\mathscr{O}}(\Delta \overrightarrow{k})\sum _{{\overrightarrow{k}}^{i}}{P}_{o}^{a}({\overrightarrow{k}}^{i}+\Delta \overrightarrow{k}){P}_{i}^{a}({\overrightarrow{k}}^{i})\exp \,[ig({\overrightarrow{k}}^{i})].$$

Note that if the optics are free from aberration and uncontrolled drift, the angular spectrum of the synthetic aperture image will simply be proportional to ${\mathscr{O}}(\Delta \overrightarrow{k})$. The term with summation is the cross-correlation of output pupil function ${P}_{o}^{a}$ and the input pupil function multiplied by uncontrolled drift ${P}_{i}^{a}\,\exp [ig]$. Because each function is complex-valued, compared to the ideal case the cross-correlation will have a smaller amplitude and stronger dependence on $\Delta \overrightarrow{k}$:

3 $$|\sum _{{\overrightarrow{k}}^{i}}{P}_{o}({\overrightarrow{k}}^{i}+\Delta \overrightarrow{k}){P}_{i}({\overrightarrow{k}}^{i})|\ge |\sum _{{\overrightarrow{k}}^{i}}{P}_{o}^{a}({\overrightarrow{k}}^{i}+\Delta \overrightarrow{k}){P}_{i}^{a}({\overrightarrow{k}}^{i})\exp [ig({\overrightarrow{k}}^{i})]|.$$

The system aberration has two major detrimental effects on the synthetic aperture image: the absolute value of the cross-correlation becomes smaller, which causes a reduction in signal intensity, and the phase value of the cross-correlation becomes dependent on the momentum difference $\Delta \overrightarrow{k}$. This distorts the PSF of the optical system, thereby distorting the target structure in the reconstructed image.

## Algorithm for aberration correction

Due to the inequality in Eq. (3), when optical system aberrations exist the total intensity of the synthetic aperture image becomes weaker than the aberration-free case. This concept is fundamental when correcting aberration in post-processing. We search for both the angle-dependent input correction function ${\theta }_{i}({\overrightarrow{k}}^{i})$ and the output correction function ${\theta }_{o}({\overrightarrow{k}}^{o})$ that maximize the total intensity of synthetic aperture image:

4 $${max}_{{\theta }_{i}({\overrightarrow{k}}^{i}),{\theta }_{o}({\overrightarrow{k}}^{o})}[{\sum }_{{\rm{\Delta }}k}{|{{\mathscr{E}}}_{SA}({\rm{\Delta }}\overrightarrow{k})|}^{2}].$$

Since it is difficult to find the correction functions ${\theta }_{i}\,\mathrm{and}\,{\theta }_{o}$ immediately, a tentative correction function ${\theta }_{i}^{(1)}({\overrightarrow{k}}^{i})$ that maximizes the total intensity of the synthetic aperture image while leaving ${\theta }_{o}=0$ is applied. This step is equivalent to placing a wavefront shaping device at the input pupil function and controlling its wavefront so as to maximize the total intensity of the reconstructed image (the graph with black bars in Fig. 2(b)). The maximization operation represented by Eq. (4) is satisfied when the cross-term due to the absolute square is real. In other words, it is satisfied when the following scalar product of the two images taken at two arbitrary incident wavevectors ${\overrightarrow{k}}_{n}^{i}$ and ${\overrightarrow{k}}_{m}^{i}$ is real.

5 $$\begin{array}{c}\sum _{\Delta \overrightarrow{k}}{ {\mathcal E} }_{{\rm{o}}}({\overrightarrow{k}}_{n}^{i}+\Delta \overrightarrow{k};\,{\overrightarrow{k}}_{n}^{i})\exp [-i{\theta }_{i}^{(1)}({\overrightarrow{k}}_{n}^{i})]\cdot { {\mathcal E} }_{{\rm{o}}}({\overrightarrow{k}}_{m}^{i}+\Delta \overrightarrow{k};\,{\overrightarrow{k}}_{m}^{i})\exp [-i{\theta }_{i}^{(1)}({\overrightarrow{k}}_{m}^{i})]\\ \,\,=\exp [i\{{\theta }_{i}^{(1)}({\overrightarrow{k}}_{m}^{i})-{\theta }_{i}^{(1)}({\overrightarrow{k}}_{n}^{i})\}]{P}_{i}^{a\ast }({\overrightarrow{k}}_{m}^{i}){P}_{i}^{a}({\overrightarrow{k}}_{n}^{i})\exp [-i\{g({\overrightarrow{k}}_{m}^{i})-g({\overrightarrow{k}}_{n}^{i})\}]\\ \,\,\sum _{\Delta \overrightarrow{k}}{[{\mathscr{O}}({\rm{\Delta }}\overrightarrow{k}){P}_{o}^{a}({\overrightarrow{k}}_{m}^{i}+\Delta \overrightarrow{k})]}^{\ast }[{\mathscr{O}}({\rm{\Delta }}\overrightarrow{k}){P}_{o}^{a}({\overrightarrow{k}}_{n}^{i}+\Delta \overrightarrow{k})].\end{array}$$

This dot product is real if ${{\rm{\theta }}}_{i}^{(1)}({\overrightarrow{k}}_{m}^{i})$ and ${{\rm{\theta }}}_{i}^{(1)}({\overrightarrow{k}}_{n}^{i})$ are set so that

6 $${\theta }_{i}^{(1)}({\overrightarrow{k}}_{m}^{i})-{\theta }_{i}^{(1)}({\overrightarrow{k}}_{n}^{i})=[{\varphi }_{i}({\overrightarrow{k}}_{m}^{i})-{\varphi }_{i}({\overrightarrow{k}}_{n}^{i})]+[g({\overrightarrow{k}}_{m}^{i})-g({\overrightarrow{k}}_{n}^{i})]+\text{arg}[{{\rm{\Phi }}}_{i}({\overrightarrow{k}}_{m}^{i};{\overrightarrow{k}}_{n}^{i})].$$

Here the term ${{\rm{\Phi }}}_{i}({\overrightarrow{k}}_{m}^{i};{\overrightarrow{k}}_{n}^{i})={\sum }_{\Delta \overrightarrow{k}}{[{\mathscr{O}}({\rm{\Delta }}\overrightarrow{k}){P}_{o}^{a}({\overrightarrow{k}}_{m}^{i}+\Delta \overrightarrow{k})]}^{\ast }[{\mathscr{O}}({\rm{\Delta }}\overrightarrow{k}){P}_{o}^{a}({\overrightarrow{k}}_{n}^{i}+\Delta \overrightarrow{k})]$ represents the error of the input correction function. If there were no output aberration, then ${{\rm{\Phi }}}_{i}({\overrightarrow{k}}_{m}^{i};\,{\overrightarrow{k}}_{n}^{i})$ is pure real and ${\rm{\arg }}\,[{{\rm{\Phi }}}_{i}({\overrightarrow{k}}_{m}^{i};\,{\overrightarrow{k}}_{n}^{i})]=0$. In such a simple case, the ${\theta }_{i}^{(1)}({\overrightarrow{k}}^{i})$ that satisfies Eq. (4) will exactly compensate ${\varphi }_{i}(\overrightarrow{k})+g({\overrightarrow{k}}^{i})$. However, the output pupil also adds aberration. Therefore, ${\rm{\arg }}\,[{{\rm{\Phi }}}_{i}({\overrightarrow{k}}_{m}^{i};\,{\overrightarrow{k}}_{n}^{i})]$ is nonzero and represents the error of the aberration correction for the input, so additional independent steps are required to correct the output aberrations. In any case, this first round of correction serves to sharpen the PSF.

After finding the first-step input correction function ${\theta }_{i}^{(1)}$, the output aberration can be estimated in a similar way, but by reversing the process. As shown in Fig. 2(c), a virtual SLM (the graph with black bars on the right in Fig. 2(c)) can be placed at the output pupil plane and control its wavefront so as to maximize the intensity of the synthetic aperture image for the case when sending the waves from the opposite side. Note that for this operation the direction of the optical axis is inverted to use the same algorithm as for the input correction operation, but that additional data measurement is not required since applying phase conjugation to the original data set can supply the equivalent information. In the phase conjugation process, the complex fields in the momentum space can be written as

7 $${ {\mathcal E} }^{pc}({\overrightarrow{k}}^{o};\,{\overrightarrow{k}}^{i}={\overrightarrow{k}}^{o}-\Delta \overrightarrow{k})={P}_{o}^{a}({\overrightarrow{k}}^{o}){{\mathscr{O}}}^{\ast }({\rm{\Delta }}\overrightarrow{k}){P}_{i}^{a}({\overrightarrow{k}}^{o}-\Delta \overrightarrow{k})\exp [ig({\overrightarrow{k}}^{o}-\Delta \overrightarrow{k})]\exp [-i{\theta }_{i}^{(1)}({\overrightarrow{k}}^{o}-\Delta \overrightarrow{k})].$$

After conducting same search process as before, the first-step correction to the output ${\theta }_{o}^{(1)}({\overrightarrow{k}}^{o})$ is estimated as

8 $${\theta }_{o}^{(1)}({\overrightarrow{k}}_{m}^{o})-{\theta }_{o}^{(1)}({\overrightarrow{k}}_{n}^{o})=[{\varphi }_{o}({\overrightarrow{k}}_{m}^{o})-{\varphi }_{o}({\overrightarrow{k}}_{n}^{o})]+\text{arg}[{{\rm{\Phi }}}_{o}({\overrightarrow{k}}_{m}^{o};\,{\overrightarrow{k}}_{n}^{o})].$$

In this case, the uncontrolled drift term is absorbed by the cross-correlation ${{\rm{\Phi }}}_{o}({\overrightarrow{k}}_{m}^{o};\,{\overrightarrow{k}}_{n}^{o}).$ This correction process preferentially corrects the output aberration such that the PSF of the illumination from output side is made sharper. With the output correction in place, the input correction was repeated and the entire process performed iteratively. Then the accumulation of the correction functions converged to its corresponding aberration maps, i.e. $\sum _{n}{\theta }_{i}^{(n)}\to {\varphi }_{i}+g$, and $\sum _{n}{\theta }_{o}^{(n)}\to {\varphi }_{o}$.

## Results

### Test target imaging

**Figure 3:**
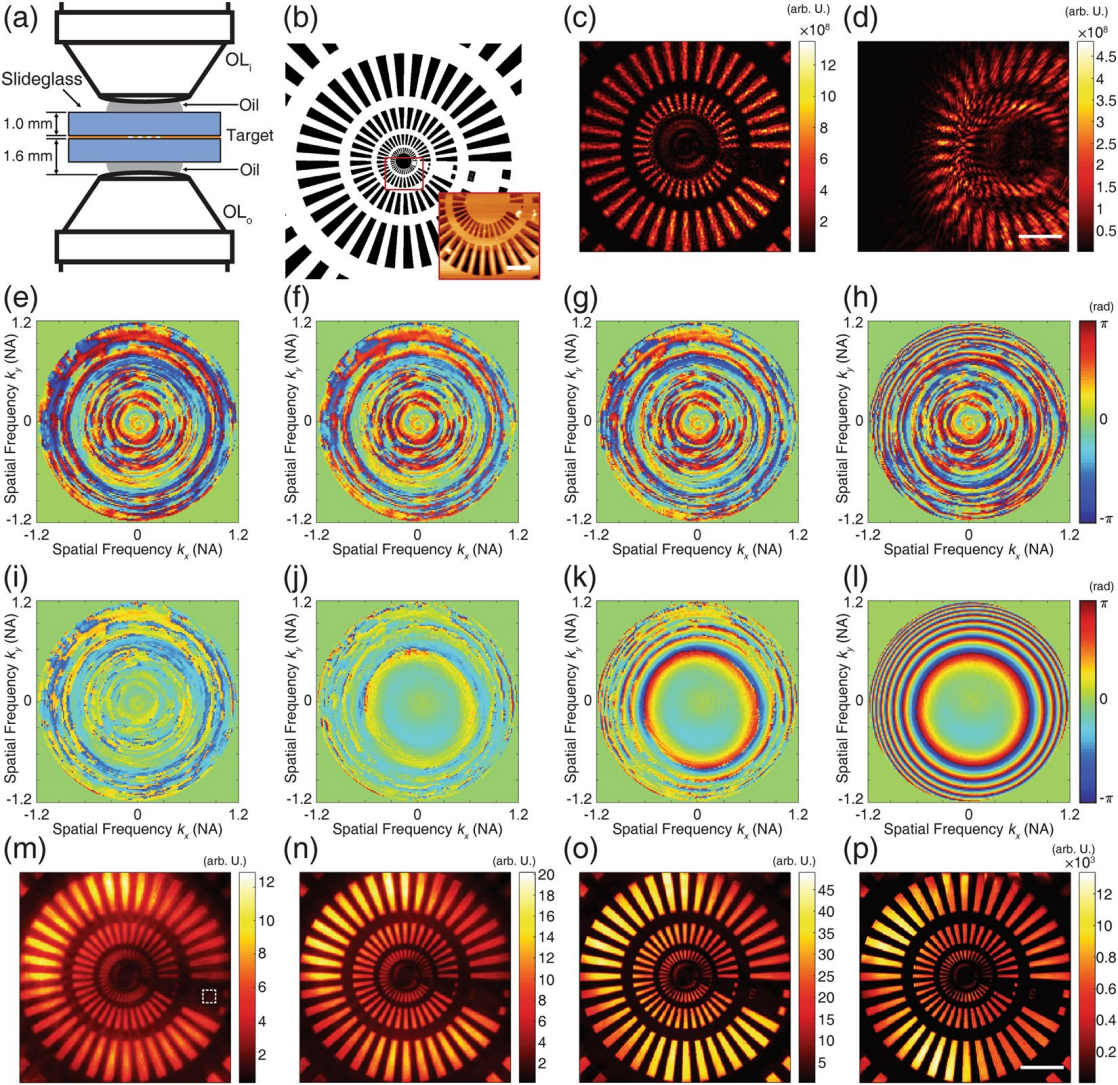
Application of CLASS algorithm to imaging a custom fabricated Siemens star resolution target. (**a**) Schematic of sample geometry. The target was fabricated on a slide glass and covered by another slide glass. Total sample thickness was about 2 mm. OL_i_: condenser for illumination, OL_o_: condenser for detection. (**b**) Diagram of the Siemens star target. Inset: an atomic force microscope (AFM) image of red squared region. Scale bar in AFM image, 2 µm. (**c**) Intensity of holographic phase image for the normal illumination. (**d**) Intensity of holographic image with oblique illumination. In this particular image, the illumination’s in-plane spatial frequency is approximately 1.0*k*_0_. For (**c**,**d**), scale bar, 10 µm. (**e**–**h**) Evolution of correction function for input aberration after first, third, fifth, and 15th iteration, respectively. Color bar, phase in radians. (**i**–**l**) Evolution of measured output aberration after the same respective numbers of iterations. (**m**–**p**) Intensity of synthetic aperture image after the same respective numbers of iterations. For each image, the color bar is normalized by the mean intensity of pixels in the white squared region in (m). Note the enhancement of maximum intensity after 15 iterations. Scale bar, 10 µm.

The correction algorithm was demonstrated by imaging a custom-made Siemens star test target. The target was fabricated on a gold film coated on a slide glass. The target was covered by a second slide glass so that the total thickness of the sample became 2 mm. The configuration of measurement is depicted in Fig. 3(a). The inset in Fig. 3(b) shows an atomic force microscope (AFM) image of the finest element marked by a red square on the main image. The central 50 × 50 μm^2^ region is imaged while scanning ${\overrightarrow{k}}^{i}$ for 19,861 different illumination angles up to 1.2 NA. The number of illumination angles depends on the size of the field of view, which is the inverse of the angular resolution, and the NA of the optical system. Figure 3(c) is a single-shot holographic image acquired with normal illumination. One can observe image distortion in the fine structures caused by the optical system’s aberration. For example, Fig. 3(d) shows the severe target structure distortion in a single-shot image taken with oblique illumination of $|{\overrightarrow{{\rm{k}}}}^{i}|=1.0{k}_{0}$.

**Figure 4:**
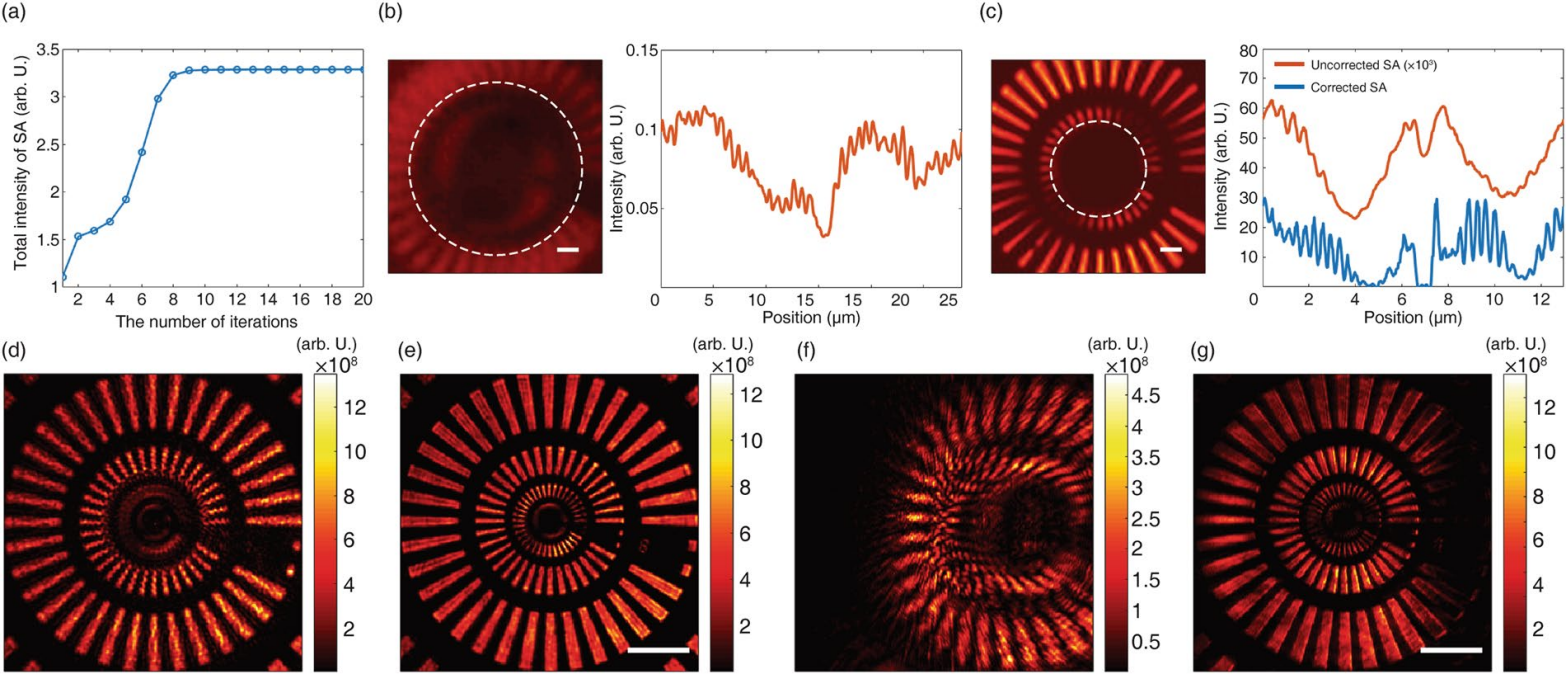
Analysis of convergence of CLASS algorithm and resolution enhancement. (**a**) Total intensity of synthetic aperture image observed during the iterative process. The total intensity converges after about ten iterations. (**b**) Left side, central 5 × 5 µm^2^ area of the synthetic aperture image before aberration correction. Scale bar, 500 nm. Right side, the line profile along the white dashed line. The periodicity of the measured pattern is 898 nm. (**c**) Left side, central 5 × 5 µm^2^ area of aberration-corrected synthetic aperture image. Right side, line profiles of the two synthetic aperture images with and without aberration correction. Scale bar, 500 nm. The line profiles are extracted from the pixels indicated by a white dashed line. The intensity of the uncorrected synthetic aperture image is multiplied by 10^3^ for better visualization. (**d**) Intensity of single-shot holographic image of the target with normal illumination. (**e**) Same image after applying the correction function for output aberration. Note that, unlike in the original image, the second finest patterns are now resolvable. (**f**) Distorted single-shot image of the obliquely illuminated target. Note that right-side region of the target has moved out of the field-of-view (FOV) due to system aberration. (**g**) By applying the same output correction function, the distortion-free image can be restored. The pattern that originally translated out of the FOV is dark. Scale bar, 10 µm.

From the set of recorded images by scanning illumination angles, we constructed ${ {\mathcal E} }_{o}({\overrightarrow{k}}^{o};\,{\overrightarrow{k}}^{i})$ and applied our aberration correction algorithm to this sets of the measured images. The input correction functions (Fig. 3(e–h)) and the output correction functions (Fig. 3(j–l)) were acquired after 1, 3, 5, and 15 rounds of iteration, respectively. Note that the input correction functions appear to be meaningless because of the coupling between input aberration and uncontrolled drift. On the other hand, the output correction functions, which are decoupled from the uncontrolled phase drift, converge to spherically symmetrical phase maps. This is a reasonable outcome considering the spherically symmetric properties of the condenser lens. The synthetic aperture images corrected by each correction function are displayed in Fig. 3(m–p). As the iterations continue, fine target structures progressively resolve. To verify the convergence of the correction algorithm, we monitored the total intensity of the synthetic aperture image at each step (Fig. 4(a)). The intensity converges to 3.5 × 10^16^ (arbitrary units) after nine or ten iterations.

The effect of aberration correction on the spatial resolution of the synthetic aperture images was investigated by observing the periodicity of a line profile. The image at the left-hand side of Fig. 4(b) shows the central 5 × 5 μm^2^ area of a synthetic aperture image without aberration correction. In this image, only uncontrolled drift and the input aberration were compensated. The line profile along the white dashed line is presented on the right-hand side of Fig. 4(b). Its periodicity was 819 nm, which is equivalent to the diffraction limit of the aberration-free optical system with 0.4 NA objective lenses. After compensating for aberration, the finest structure of the target becomes resolvable as shown in the left-hand side of Fig. 4(c). Two line profiles along the white dashed line are obtained from the synthetic aperture images with (blue curve) and without (red curve) aberration correction. Note that the intensity of the line profile of the uncorrected image was multiplied by 10^3^ for better visualization. Although there was slight dependence on the direction of the pattern, the structure of 372 nm periodicity was resolved with aberration correction. Thus, spatial resolution was enhanced from 819 nm to 372 nm. Considering that the diffraction-limit spatial resolution $\Delta =\frac{\lambda }{({{\rm{NA}}}_{ill}+{{\rm{NA}}}_{col})}=327\,{\rm{nm}}$ in our system, this result confirms that our aberration correction algorithm almost perfectly compensates for system aberration and allows microscope condensers to be used as objective lenses.

The detrimental effect of the system aberration and the result of the aberration correction can be observed from the single shot images. Figure 4(d) is an uncorrected image captured for the normal illumination. While the third and fourth groups of patterns were resolvable, the second and the first groups remained unresolved. After applying the output correction function obtained from the CLASS algorithm, the second finest patterns became resolvable (Fig. 4(e)). Here, the input correction function needs not be applied because the sample was illuminated with a plane wave of a single illumination angle.

The difference between the images with and without the application of the correction function was more pronounced for the case of oblique illumination. In this case, the target was illuminated with a plane wave of a lateral momentum $\approx 1.0{k}_{0}$. Without output correction, the single-shot image (Fig. 4(f)) was more severely distorted than that of the normal illumination. Because of the oblique illumination, relatively large portion of the scattered light propagated through the outer region of the output pupil where the aberration was steep. However, the target structure was retrieved with the output correction function in place (Fig. 4(g)). These results support the reliability of the CLASS algorithm.

### Yeast cell imaging

**Figure 5:**
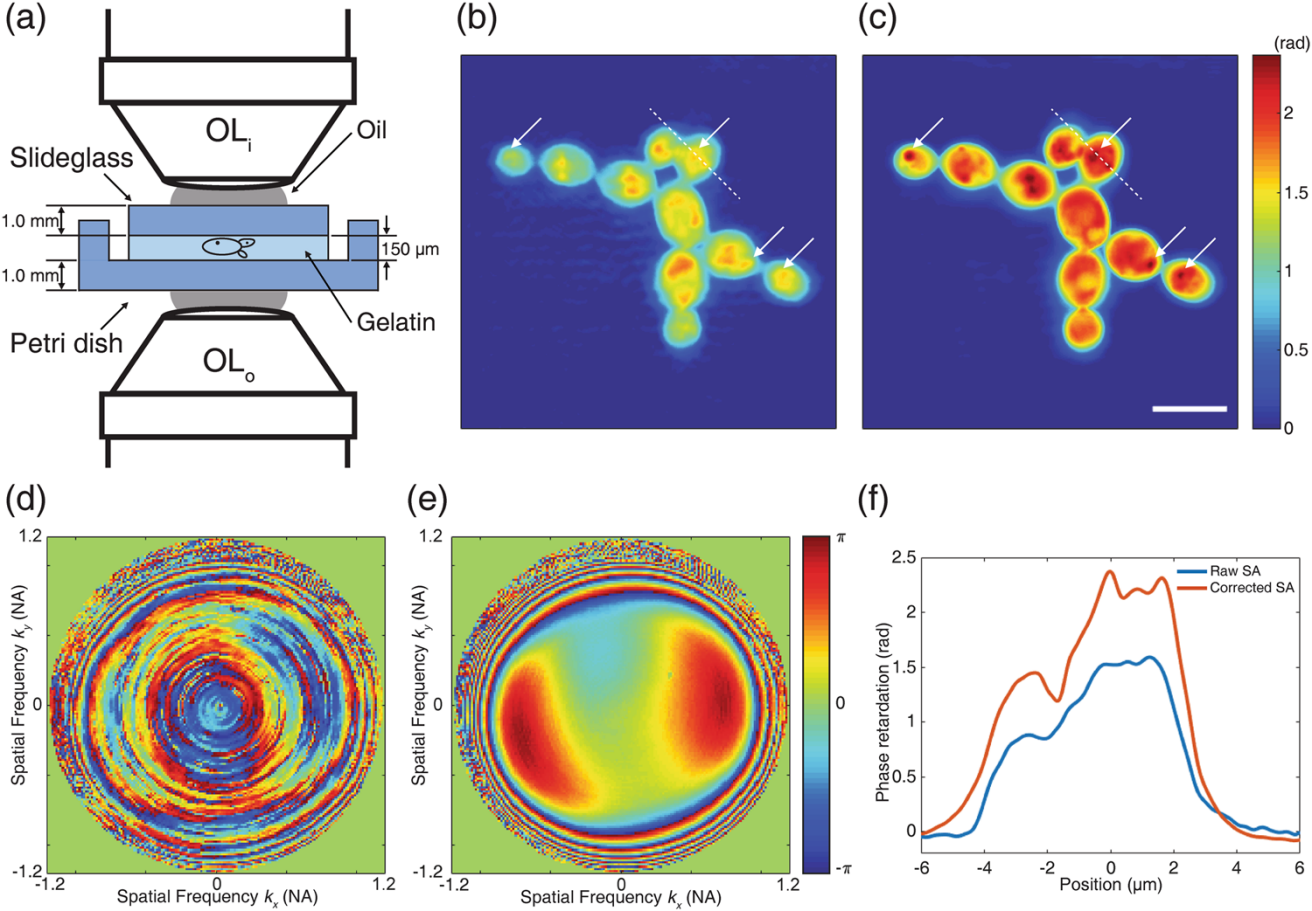
Phase contrast image of yeasts with *in-situ* aberration correction. (**a**) Schematic of the sample geometry. Yeast cells were embedded in a gelatin block of 150 µm thickness. The block is placed on a petri dish and covered by a slide glass. The thicknesses of the dish bottom and the slide glass are both 1 mm. OL_i_: condenser for illumination, OL_o_: condenser for detection. (**b**) Phase map of uncorrected synthetic aperture image. (**c**) Phase map of aberration-corrected synthetic aperture image. Scale bar, 10 µm. Color bar, phase in radians. We used the same color bar for these two images. (**d–e**) Measured correction functions for the input and output aberration. Color bar, phase in radians. (**f**) Line profiles of phase retardation along the white dashed lines in (**b**) and (**c**).

We applied the developed method to the imaging of biological cells. We prepared budding yeast *Saccharomyces cerevisiae* (*S. cerevisiae*) which was purchased from the Korean collection for type cultures (KCTC, Korea). The yeast cells were mixed with a 3% gelatin solution (G1393, Sigma) and then poured into a Petri dish (10090, SPL; bottom thickness ≈ 1 mm) to make a gelatin block of about 150 μm thickness at 4 °C. The block was covered by a slide glass, making a total sample thickness of 2.1 mm. This was too thick for an ordinary oil-immersion objective lens to reach the sample. The geometry of the sample is illustrated in Fig. 5(a).

First, the synthetic aperture image was computed without aberration correction (Fig. 5(b)). From the same dataset, the optical system’s aberration including sample-induced aberration was measured by optimizing the total intensity of the synthetic aperture image. Figure 5(c) shows the aberration-corrected phase map of the yeast cells after ten rounds of iteration. The corresponding input and output correction functions are plotted in Fig. 5(d,e), respectively. Unlike the image reconstructed without aberration correction, in the aberration-corrected image granular structures within the cells were clearly visible (see white arrows in Fig. 5(b,c)). Figure 5(f) presents the phase retardation along the white dashed lines in each of the phase images, and shows that the spatial phase variation was sharpened by aberration correction.

The input correction function in Fig. 5(d), like the functions measured from the Siemens star target, has a complicated structure because of uncontrolled phase drift. On the other hand, the output correction function for the yeast cell sample shown in Fig. 5(e) has a different structure to that of the Siemens star dataset. This highlights the effect of the sample-induced aberration caused by the petri dish, which has a different refractive index from the glass.

### Separation of the input aberration and the uncontrolled drift

The input and output aberration correction maps identified by our algorithm are presented in Fig. 3(e–l) and Fig. 5(d–e). At first glance, the output correction maps were accurately measured, as judged by their circularly symmetrical structure. The condenser lens for detection presents strong aberration, especially at NA higher than 0.8, suggesting the likelihood of the pronounced degradation of spatial resolution. However, the input correction map presented rather irregular patterns and looked quite different from the output correction map despite the same model of condenser lens being used. In fact, this is due to uncontrolled phase drift during measurement. As discussed in Eq. (6), ${\theta }_{i}({\overrightarrow{k}}^{i})$ converges to ${\varphi }_{i}({\overrightarrow{k}}^{i})+g({\overrightarrow{k}}^{i})$. The mixing of aberration and uncontrolled drift is not an issue for the present study since the correction of their summation is sufficient for the reconstruction of synthetic aperture images. But the separation of other scanning-type microscopes such as confocal or two-photon microscopes in such a way that our method provides aberration map to be corrected for the focus scanning microscopes.

**Figure 6:**
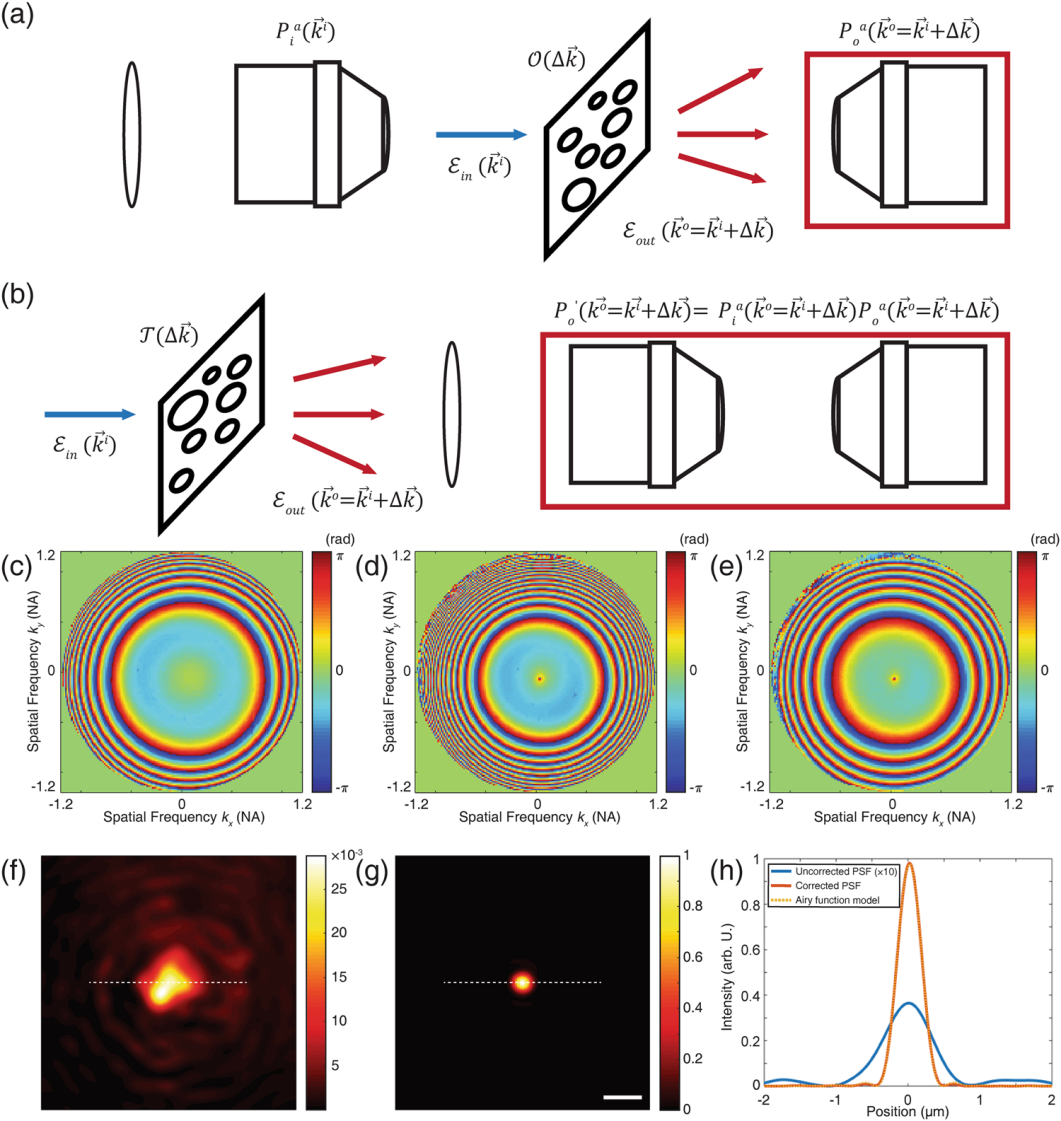
Separation of input aberration and uncontrolled drift and the effect of input aberration on PSF. (**a**) Ordinary measurement configuration. The target is placed at the sample plane. The measured output aberration is equal to the aberration of the imaging condenser (marked by the red box). (**b**) A test target is placed at the image plane before the illumination condenser lens. The measured output aberration in this configuration corresponds to the combined aberration of the two condenser lenses. (**c**) Measured output aberration by placing the target at the sample plane, i.e., the configuration in (**a**). (**d**) Measured output aberration from a test target placed at the image plane before the illumination condenser, i.e. the configuration in (**b**). (**e**) Subtracted aberration map, (**c**) from (**d**). This corresponds to the input aberration originating from the illuminating condenser lens. (**f**) Intensity of PSF simulated from the transmission matrix with input aberration in place. To single out the effect of input aberration, only uncontrolled drift and output aberration are corrected numerically. (**g**) Intensity of PSF simulated from the aberration-free transmission matrix. Scale bar, 1 µm for both images. The two images are normalized to make the maximum intensity of aberration-free PSF unity. (**h**) Line profiles along the white dashed lines in the PSF images. The intensity of the PSF with input aberration is amplified 10-fold for better visualization. The PSF with input aberration correction is well described by an Airy function model (yellow dashed line). Note that the peak intensity is enhanced 34.7 times (inverse of Strehl ratio) by correcting for input aberration.

This uncontrolled drift cannot be separated from input aberration without additional measurements. To address this issue, we propose a way to separate the input aberration from uncontrolled drift by measuring one more set of images, which contain the combined aberration of the two condensers. Firstly, the two correction functions are computed from the target images at the sample plane. The output correction function ${\theta }_{o}({\overrightarrow{k}}^{o})={\varphi }_{o}({\overrightarrow{k}}^{o})$ compensates for output aberration alone as shown in Fig. 6(a). In contrast, the input correction function ${\theta }_{i}({\overrightarrow{k}}^{i})={\varphi }_{i}({\overrightarrow{k}}^{i})+g({\overrightarrow{k}}^{i})$ corresponds to the sum of input aberration and uncontrolled drift. One more set of images was then taken by placing a test target at the conjugate image plane before the input condenser lens (Fig. 6(b)). In this configuration, we can consider the system as having no input aberration and a stronger output aberration than before as the two condensers’ pupil functions are multiplied. Therefore, the complex field ${ {\mathcal E} ^{\prime} }_{SA}$ for the synthetic aperture can be written as ${ {\mathcal E} ^{\prime} }_{SA}(\Delta \overrightarrow{k})={\mathscr{O}}^{\prime} ({\rm{\Delta }}\overrightarrow{k})\sum _{{\overrightarrow{k}}^{i}}{P}_{o}^{a}({\overrightarrow{k}}^{i}+{\rm{\Delta }}\overrightarrow{k}){P}_{i}^{a}({\overrightarrow{k}}^{i}+{\rm{\Delta }}\overrightarrow{k})\exp [i(g-g^{\prime} )({\overrightarrow{k}}^{i})],$ where ${\mathscr{O}}^{\prime} ({\rm{\Delta }}\overrightarrow{k})$ describes the angular spectrum of the test target. In this case, the correction functions of our aberration correction algorithm will yield ${\theta ^{\prime} }_{i}({\overrightarrow{k}}^{i})=\exp [i(g-g^{\prime} )({\overrightarrow{k}}^{i})]$, and ${\theta ^{\prime} }_{o}({\overrightarrow{k}}^{o})=\text{arg}[{P}_{o}^{a}({\overrightarrow{k}}^{o}){P}_{i}^{a}({\overrightarrow{k}}^{o})]=$ ${\varphi }_{o}({\overrightarrow{k}}^{o})+{\varphi }_{i}({\overrightarrow{k}}^{o})$, respectively. Therefore, the aberration of the input condenser ${\varphi }_{i}$ can be computed from ${\theta ^{\prime} }_{o}({\overrightarrow{k}}^{o})-{\theta }_{o}({\overrightarrow{k}}^{o})$. Figure 6(c–e) show the experimental results of ${\theta }_{o}({\overrightarrow{k}}^{o})={\varphi }_{o}({\overrightarrow{k}}^{o})$, ${\theta ^{\prime} }_{o}({\overrightarrow{k}}^{o})={\varphi }_{i}+{\varphi }_{o}({\overrightarrow{k}}^{o})$, and ${{\rm{\theta }}^{\prime} }_{{\rm{o}}}-{{\rm{\theta }}}_{o}({\overrightarrow{k}}^{o})={\varphi }_{i}({\overrightarrow{k}}^{i})$, respectively. Note that the input and output correction functions have different structures, as expected from the transmission geometry.

To verify whether the input aberration map identified in this way is correct, we separated the three correction functions ${\varphi }_{i}({\overrightarrow{k}}^{o})$, ${\varphi }_{o}({\overrightarrow{k}}^{o})$, and $g({\overrightarrow{k}}^{i})$. We then numerically compensated for the output aberration ${\varphi }_{o}({\overrightarrow{k}}^{o})$ and the uncontrolled drift $g({\overrightarrow{k}}^{i})$ on the images, and computed PSF at the sample plane (Fig. 6(f)). In this case, PSF distortion is solely due to the aberration ${\varphi }_{i}$ caused by the input condenser. The correction for ${\varphi }_{i}$ was then additionally applied to form the PSF shown in Fig. 6(g). The resulting width of the PSF in Fig. 6(g) is much narrower than that in Fig. 6(f). From the line profiles along the white dashed lines (Fig. 6(h)), we could acquire the FWHMs of the uncorrected and corrected PSFs, which were 770 nm and 444 nm, respectively. The shape of the focus became an Airy function pattern after correcting for input aberration. We confirm this with a curve fitting of an Airy function model (yellow dashed line in Fig. 6(h)). The squared norm of the residual, which quantifies the variation of the data from the model, is 2.5 × 10^−3^. Note that the intensity of the uncorrected PSF was multiplied by ten for better visualization and that the peak intensity was enhanced 34.7 times by compensating for input aberration.

## Discussion and Conclusion

In this study, we developed a method to measure and correct the aberration of an optical system in both its illumination and detection paths. By using this method, a pair of commercial microscope condensers turned into diffraction-limited objectives with an order-of-magnitude longer working distance than the conventional oil-immersion objective lenses. This allowed us to image specimens prepared between thick slide glasses with a spatial resolution of approximately 372 nm, a value close to the diffraction limit spatial resolution set by 1.2 NA at the wavelength of 785 nm. Furthermore, we separately identified the aberrations of the illumination and collection lenses. In particular, we could separate out the uncontrolled phase drift innate to coherent imaging from the input aberration to exclusively identify the aberration of the input condenser lens.

Relatively slow data acquisition speed is one of the current technical limitations. To capture a synthetic aperture image, one needs to capture multiple wide-field holographic images. The required number of images, although it depends on the field of view and the numerical aperture, is 19,861 in our measurements if one wants to sample the illumination angles fully. In our experiment, the camera frame rate was 20 fps such that it takes about 1,000 s for a set of measurements. Although this is not a critical issue for the present study where the characterization of the imaging system’s aberration is a major concern, there is room to shorten the data acquisition time. Since there are plenty of photons to make use of in the transmission measurement, we can use a high-frame-rate camera to speed up the measurement. The number of images required for the aberration correction can also be reduced depending on the angular variation of aberrations, which will further enhance overall speed of the proposed method.

The proposed method ameliorates the image quality in the presence of steeply varying aberration at the pupil planes, which typically occurs with lenses of a large diameter, by the fine stepping of angular scanning and post-processing of the acquired set of coherent images. In comparison with the correction methods using feedback control and wavefront shaping devices^29,31^, the proposed method can be faster, especially when a large number of the correction modes matters. Even at the current implementation of slow image acquisition, we could correct 20 different angular modes per second. This is equivalent to correcting the same number of Zernike modes per second, which is high speed in the conventional adaptive optics. In the present study, we restricted our interest to deal with optical system’s aberrations in transmission geometry, but the algorithm would be equally valid for correcting sample-induced aberrations as we have demonstrated in the reflection geometry. Since elastic scattering, not fluorescence, is used for aberration correction, the light intensity level is low enough to induce photo-bleaching. Therefore, our technique can be effectively combined with fluorescence-based imaging, by providing aberration correction maps and helping to increase the working distances at which high spatial resolution can be maintained.

## Methods

### Data processing

Custom scripts by Matlab R2016b (MathWorks Inc.) were used to CLASS optimization to the acquired set of images was conducted by using on a desktop computer (Intel Core i7-6700K CPU, 4.0 GHz, 64 GB RAM). A set of raw interference images were converted into complex fields with amplitude and phase by applying 2D Hilbert transformation. Hilbert transformation of 19,861 images takes 498 s. From these image sets, we constructed ${{\mathscr{E}}}_{o}({\overrightarrow{k}}^{o};{\overrightarrow{k}}^{i})$, which took 595 s. After that, the CLASS optimization process takes 440 s per iteration. The required number of iterations varies depending on the target objects. The iterative process was terminated by monitoring convergence of total synthetic aperture intensity. Including 15 iterations of CLASS optimization, it takes 8,289 s to reconstruct an aberration-free SA image from raw images.

### 2D Hilbert transform

2D Hilbert transform is a process that reconstructs the phase and amplitude maps of a sample beam from an off-axis hologram. The transform consists of Fourier transform, low-pass filtering, and shift of the spectrum. To acquire an off-axis hologram, a reference beam of lateral spatial frequency ${\overrightarrow{k}}^{R}$ is introduced at the detector. The camera measures the intensity map, $I={|{E}_{S}+{E}_{R}|}^{2}$, where *E*_*S*_ and *E*_*R*_ are the sample and reference waves, respectively. The off-axis hologram can be decomposed into three different spectra centered at $\overrightarrow{0}$, ${\overrightarrow{k}}^{R}$, and $-{\overrightarrow{k}}^{R}$ in the Fourier space. The first one is an autocorrelation of the sample complex field (Fourier transform of sample intensity). The second and the third terms are the spatial frequency spectrum of *E*_*S*_ and its complex conjugate, respectively. The circular spectrum centered at ${\overrightarrow{k}}^{R}$ is selected by low-pass filtering with the bandwidth (the radius of the circular low-pass filter) set by the field of view and numerical aperture, and shifted by $-{\overrightarrow{k}}^{R}$ to eliminate the carrier frequency. By taking the inverse Fourier transform of the resulting spectrum, we could obtain the complex field map of *E*_*S*_, which completes the 2D Hilbert transform.
